# Our Journal for 1878

**Published:** 1878-01-20

**Authors:** 


### *EDITORIAL AND MISCELLANEOUS.*

Our Journal for 1878.—The type of the present volume of our journal is changed, we think for the better, but not reduced in size as was contemplated. In lieu of this, we have added four pages of reading matter, giving about fifty additional pages for the year. While this and other additional expenses have been incurred for the improvement of the Journal and the benefit of our subscribers, the same low rate of subscription will be continued. We trust that these improvements and sacrifices will be appreciated by our readers.

